# Improving Tobacco Cessation Programs in Lao People's Democratic Republic: Perspectives of Healthcare Professionals

**DOI:** 10.1200/GO.22.30000

**Published:** 2022-05-05

**Authors:** Phonepadith Xangsayarath, Shweta Kulkarni, Dalouny Xayavong, Chanthavy Soulaphy, Khatthanaphone Phandouangsy, Phayvanh Keopaseuth, Khamsing Keothongkou, Tina Le, Damon Vidrine, Jennifer Vidrine, Michael Businelle, Thanh Bui

**Affiliations:** ^1^ National Center for Laboratory and Epidemiology, Ministry of Health of Lao PDR, Vientiane, Laos; ^2^ Hudson College of Public Health, University of Oklahoma Health Sciences Center, Oklahoma City, OK; ^3^ Secretariat of the National Tobacco Control Committee of Lao PDR, Vientiane, Laos; ^4^ Setthathirath Hospital, Ministry of Health of Lao PDR, Vientiane, Laos; ^5^ Champasak Hospital, Ministry of Health of Lao PDR, Vientiane, Laos; ^6^ TSET Health Promotion Research Center, Stephenson Cancer Center, University of Oklahoma Health Sciences Center, Oklahoma City, OK; ^7^ Moffitt Cancer Center, Tampa, FL

## Abstract

Tobacco use represents a major public health problem in Lao People's Democratic Republic (Lao PDR), however very few tobacco cessation programs are available. This formative, qualitative study of Lao healthcare professionals (HCP) aims to elicit both factors that help smokers quit and mechanisms to improve the effectiveness of tobacco treatment programs.

**METHODS:**

We conducted in-depth interviews with 28 HCPs at two hospitals, Setthathirath Hospital in Vientaine Capital and Champasak Hospital in Champasak Province. We used purposive sampling to select a diverse sample with regard to age, sex, work position, and clinical speciality. Interviews were voice recorded and transcribed verbatim. Data were analyzed using thematic content analysis aided by the MAXQDA program. Codes and themes related to smoking cessation behavior were based on theoretical constructs of the Phase-Based Model (PBM). Codes and themes related to intervention implementation or dissemination were based on the Practical, Robust Implementation and Sustainability Model (PRISM).

**RESULTS:**

Regarding PBM-based factors to help smokers quit, HCPs emphasized the need for medications to address craving, repeating quit-smoking advice, arranging follow-ups to monitor patients' progress, and advising patients on exercising or other distracting activities to cope with urges and stress. HCPs also identified visual education tools (e.g., short videos or images) as helpful, and that emphasis of smoking-related harms to patients' families increased smoker's motivation to quit. Salient PRISM-based factors for program implementation include the continuing dissemination of information regarding smoking-related harms in mass media, highlighting successful quit stories, the need for HCPs to be a model of becoming non-smokers, and the local enforcement of tobacco control policies (e.g., smoke-free environments).

**CONCLUSION:**

This study identified several areas for improvement to increase the likelihood of success for tobacco treatment programs in Lao PDR, including individual-level (smokers') factors, provider-level factors, and external environment factors.

